# Spirit-Quieting Traditional Chinese Medicine May Improve Survival in Prostate Cancer Patients with Depression

**DOI:** 10.3390/jcm8020218

**Published:** 2019-02-08

**Authors:** Po-Hung Lin, Shun-Ku Lin, Ren-Jun Hsu, See-Tong Pang, Cheng-Keng Chuang, Ying-Hsu Chang, Jui-Ming Liu

**Affiliations:** 1 Division of Urology, Department of Surgery, Chang Gung Memorial Hospital at Linkou, Taoyuan 333, Taiwan; m7587@adm.cgmh.org.tw (P.-H.L.); jacobpang@cloud.cgmh.org.tw (S.-T.P.); chuang89@cgmh.org.tw (C.-K.C.); anatomy@cloud.cgmh.org.tw (Y.-H.C.); 2 Graduate Institute of Clinical Medical Science, College of Medicine, Chang Gung University, Taoyuan 333, Taiwan; 3 Department of Chinese Medicine, Taipei City Hospital, Ren-Ai Branch, Taipei 106, Taiwan; gigilaskl@gmail.com; 4 Institute of Public Health, National Yang-Ming University, Taipei 112, Taiwan; 5 Graduate Institute of Life Sciences, National Defense Medical Center, Taipei 114, Taiwan; hsurnai@gmail.com; 6 Cancer Medicine Center of Buddhist Hualien Tzu Chi Hospital, Tzu Chi University, Hualien 970, Taiwan; 7 Department of Pathology and Graduate Institute of Pathology and Parasitology, Tri-Service General Hospital, National Defense Medical Center, Taipei 114, Taiwan; 8 Division of Urology, Department of Surgery, Taoyuan General Hospital, Ministry of Health and Welfare, Taoyuan 330, Taiwan; 9 Department of Medicine, National Yang-Ming University, Taipei 112, Taiwan

**Keywords:** prostate cancer, depression, traditional Chinese medicine

## Abstract

Depression is associated with higher mortality in prostate cancer. However, whether traditional Chinese medicine (TCM) for depression improves outcomes in patients with prostate cancer is unclear. This retrospective cohort study evaluated the association between TCM for depression and mortality in patients with prostate cancer. During the period 1998–2012, a total of 248 prostate cancer patients in Taiwan with depression were enrolled and divided into three groups: TCM for depression (*n* = 81, 32.7%), TCM for other purposes (*n* = 53, 21.3%), and no TCM (*n* = 114, 46.0%). During a median follow-up of 6.2 years, 12 (14.8%), 13 (24.5%), and 36 (31.6%) deaths occurred in the TCM for depression, TCM for other purposes, and no TCM groups, respectively. After adjusting age at diagnosis, urbanization, insured amount, comorbidity disease, and prostate cancer type, TCM for depression was associated with a significantly lower risk of overall mortality based on a multivariate-adjusted Cox proportional-hazards model (hazard ratio 0.42, 95% confidence interval: 0.21–0.85, *p* = 0.02) and Kaplan–Meier survival curve (log-rank test, *p* = 0.0055) compared to no TCM. In conclusion, TCM for depression may have a positive association with the survival of prostate cancer patients with depression.

## 1. Introduction

Prostate cancer is the most common male urological cancer worldwide. It is the 5th leading cancer in terms of incidence and the 7th leading cancer in terms of mortality in Taiwan [1]. The clinical course of prostate cancer is longer compared to other malignancies [2]. Therefore, other predisposing factors which may worse patients’ mental or physical health are warranted to investigate to improve the quality of life of longer living prostate cancer patients.

Depression is associated with higher mortality risk for patients with prostate cancer, and particularly for those with metastatic prostate cancer. Prostate cancer patients with depression are associated with a higher risk of mortality [3,4,5]. Some studies have shown that intervention for depression may increase patients’ adherence and reduce the mortality of cancer. However, these studies have focused mainly on western types of antidepressants or psychotherapies [6], and investigations on traditional and alternative medicines for depression in patients with cancer are lacking.

More than 20% of patients with prostate cancer receive traditional Chinese medicine (TCM) as an adjunct to conventional treatment in Taiwan [7]. Many traditional Chinese herbal medicines have the potential to treat prostate cancer. For example, *Rhizoma Curcumae Longae* can inhibit cancer cell growth and reduce the expression of vascular endothelial growth factor [8,9]. In addition, clinical trials have shown that Kamikihito can improve fatigue and anxiety in patients with prostate cancer by restoring the balance of the autonomic nervous system [10]. However, whether TCM for depression improves the outcomes of patients with prostate cancer remains unclear. The aim of this study was to evaluate the association between TCM for the treatment of depression and overall mortality in patients with prostate cancer.

## 2. Materials and Methods

### 2.1. Database

We used the Longitudinal Health Insurance Database 2000 (LHID2000) to conduct this retrospective cohort study. The LHID2000 is derived from the National Institutes of Health, which sampled a million representative patients from all recipients of National Health Insurance in the year 2000 with a sample area comprising Taiwan and its affiliated islands. No significant difference exists between the sampled and original population in terms of sex, age, or insurance coverage.

The National Institutes of Health documented all medical information of the sampled patients, including diagnosis, prescription, hospital records, and medical expenses from 1997 to 2012. The LHID2000 database is part of the National Health Insurance Research Database, which has two advantages: complete long-term tracking and a sampling range covering more than 99% of the population, making it ideal for long-term cancer tracking research [11]. The Research Ethics Committee of Chang Gung Memorial Hospital reviewed the research protocol and approved this research (103-2084B).

### 2.2. Study Population

**Figure 1:**
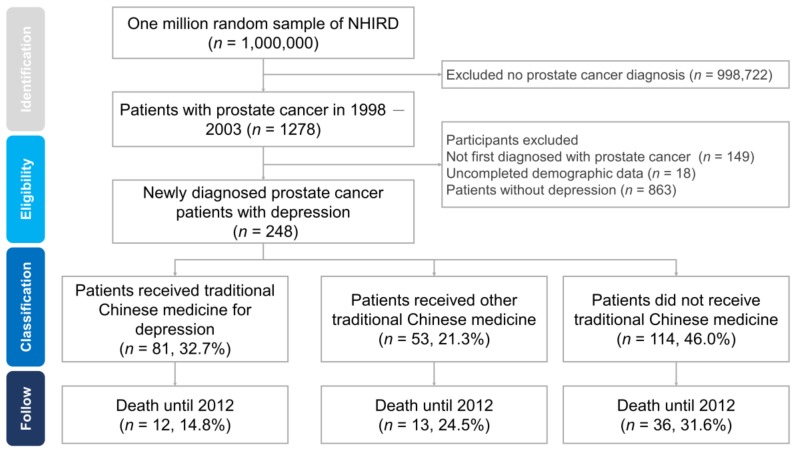
Recruitment flowchart of prostate cancer patients with depression. We enrolled 248 prostate cancer patients with depression and divided participants into three groups: patients who received traditional Chinese medicine (TCM) for depression (*n* = 81, 32.7%), patients who received other TCM (*n* = 53, 21.3%), and patients who did not receive TCM (*n* = 114, 46.0%). The number of deaths was 12 (14.8%), 13 (24.5%), and 36 (31.6%) in patients who received TCM for depression, patients who received other TCM, and patients who did not receive TCM, respectively.

We included patients with newly diagnosed prostate cancer between 1998 and 2003 and followed the cohort to 2012. Prostate cancer diagnoses were identified based on an International Classification of Diseases, 9th revision, Clinical Modification (ICD-9-CM) code of 185 [12], and diagnoses of depression were identified based on ICD-9-CM codes of 296.2, 296.3, 296.5, 296.8, 300.4, 309.0, 309.1, 311, 648.4, and 780.7. All prostate cancer patients received catastrophic illness certificates, which are subject to government review and enable medical fee reductions. Depression was diagnosed by a psychiatrist and treated with antidepressants. We employed the following exclusion criteria: participants who had the diagnosis of prostate cancer before the study period, those with incomplete demographic data, and those without depression. Figure 1 shows the recruitment flowchart for prostate cancer patients with depression.

### 2.3. TCM

National Health Insurance in Taiwan is one of the few national insurance systems that cover TCM. The LHID2000 contains complete TCM information, including prescription details, such as drug name, dosage, dosage form, duration, and method of administration. We assessed the LHID2000 files on ambulatory care expenditure by visits and details of ambulatory care orders, and classified patients who received TCM treatment after the diagnosis of prostate cancer into the “TCM users” group. These methods have been widely used in TCM database research.

After analyzing the TCMs used by prostate cancer patients with depression, we identified the following six formulae for the treatment of depression: Wen-Dan-Tang (溫膽湯) [13], Tian-Wang-Bu-Xin-Dan (天王補心丹) [14], Suan-Zao-Ren-Tang (酸棗仁湯) [15], Jia-Wei-Xiao-Yao-San (加味逍遙散) [16], Gan-Mai-Da-Zao-Tang (甘麥大棗湯) [17], and Chai-Hu-Jia-Long-Gu-Mu-Li-Tang (柴胡加龍骨牡蠣湯) [18]. We also used the diagnosis of a TCM practitioner to confirm the indications for the aforementioned formulae. We classified patients who took these compounds into the “TCM for depression (or spirit-quieting TCM)” group, and the rest of the patients were classified into the “other TCM therapy” group. We calculated the type, dose, and duration of each TCM taken by the patients.

### 2.4. Study Outcomes

All-cause mortality was the primary outcome of this study. We obtained all of the patients’ hospitalization data (from the inpatient admissions file) and insurance information (from the registry for beneficiaries file) from the LHID2000. In this study, mortality was defined as a patient being discharged from hospital because of death and removed from the universal health insurance system, which is a method that has been widely used in health insurance research. We defined the follow-up duration from the start of a diagnosis of prostate cancer to the death of the patient or the end of the study (December 31, 2012). However, to avoid immortal time bias, we adjusted the tracking period of the TCM treatment group. The time before the start of TCM treatment was not calculated to avoid under- or over-estimation of the overall survival.

### 2.5. Adjustment of Covariates

In past studies, many comorbidities have been found to be associated with TCM use and prostate cancer mortality, including diabetes mellitus (ICD-9-CM: 250), coronary heart disease (ICD-9-CM: 410–414), chronic kidney disease (ICD-9-CM: 585, 586, and 588), heart failure (ICD-9-CM: 428), cerebral vascular disease (ICD-9-CM: 430–438), liver cirrhosis (ICD-9-CM: 571), and hypertension (ICD-9-CM: 401–405). We used the Cox regression model to correct for and reduce the confounding effects of these diseases in the study population. 

We also included four demographic variables to compare differences between the groups, including the age at which prostate cancer was diagnosed, the degree of urbanization in the insurance area, and the level of insurance coverage (which can approximately represent household income). Although the database does not contain staging data for cancer, we can estimate the type of prostate cancer from the treatment received by the patient. Radical prostatectomy or radiation therapy indicates that the patient has localized or locally advanced prostate cancer, androgen deprivation therapy indicates metastatic prostate cancer, and chemotherapy indicates castration-resistant cancer.

### 2.6. Statistical Analysis

We assessed demographic variables and medical characteristics (comorbidity disease and treatment of prostate cancer) to determine differences among three groups: patients who received TCM for depression, patients who received other TCM therapy, and patients who did not receive TCM. We used chi-square tests to assess differences among the groups. We used the Cox proportional-hazards regression model to measure the risk of death in prostate cancer and calculated the adjusted hazard ratios and 95% confidence intervals (CIs). We also plotted Kaplan–Meier curves and calculated the *p*-values of log-rank tests to determine the relationship between overall mortality and time for different groups. We used the SAS statistical software package (version 9.4, SAS Institute, Cary, NC, USA) for data processing and statistical analysis. 

## 3. Results

### 3.1. Baseline Characteristics

We enrolled 248 prostate cancer patients with depression and further divided them into three groups according to TCM use: patients who received TCM for depression (spirit-quieting TCM group) (*n* = 81, 32.7%), patients who received other TCM therapy (*n* = 53, 21.3%), and patients who did not receive TCM (*n* = 114, 46.0%). The baseline demographic and medical characteristics of the study patients are shown in Table 1. Most patients with prostate cancer who participated in the study were elderly, and more than 60% were older than 70 years. We assessed the distribution of patients based on age, insurance coverage, and the degree of urbanization in the insurance area. The chi-square results showed no differences among the patients who received spirit-quieting TCM, other TCM therapy, and no TCM treatment. The most common type of prostate cancer was metastatic cancer, followed by localized or locally advanced cancer and castration-resistant cancer. No significant differences were observed in the distribution of prostate cancer and TCM treatment. 

We analyzed the relationships between the different TCM treatment groups and comorbid diseases, including diabetes mellitus, chronic kidney disease, cerebrovascular disease, coronary heart disease, heart failure, liver cirrhosis, and hypertension. The patients who received spirit-quieting TCM had higher comorbidities, except for liver cirrhosis. 

### 3.2. Longitudinal Analysis of Mortality Risk of Prostate Cancer

During a median follow-up period of 6.2 years, the number of deaths was 12 (14.8%), 13 (24.5%), and 36 (31.6%) among the patients who received TCM for depression, those who received other TCM therapy, and those who did not receive TCM, respectively. 

**Figure 2:**
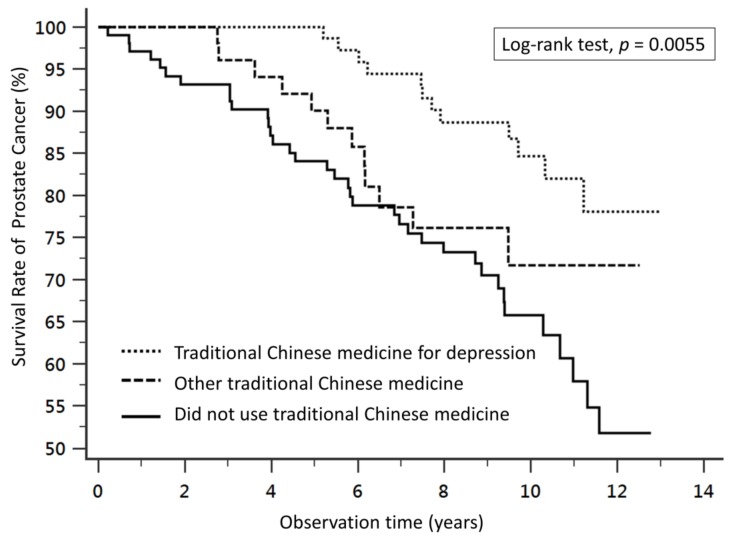
Survival curves of prostate cancer patients with different treatments. Kaplan–Meier survival curves and log-rank analyses revealed significant differences in the survival rates between patients who received traditional Chinese medicine (TCM) for depression, patients who received other TCM, and patients who did not receive TCM (log-rank test, *p* = 0.0055).

Compared to patients who did not receive TCM, TCM for depression was associated with a lower risk of overall mortality in a multivariate-adjusted Cox proportional-hazards model and Kaplan–Meier survival curve analysis (log-rank test, *p* = 0.0055) (Figure 2). The adjusted hazard ratio (aHR) of the patients who received TCM for depression was significantly lower at 0.42 (95% CI: 0.21–0.85, *p* = 0.02) than the patients who did not receive TCM (Table 2).

## 4. Discussion

To the best of our knowledge, this is the first study to investigate the use of spirit-quieting TCM therapy and the risk of death in patients with prostate cancer and depression. We included 248 patients with depressive prostate cancer for an average of 6.2 years of follow-up. We found that TCM for depression (including at least one of the following spirit-quieting TCM formulae: Wen-Dan-Tang, Tian-Wang-Bu-Xin-Dan, Suan-Zao-Ren-Tang, Jia-Wei-Xiao-Yao-San, Gan-Mai-Da-Zao-Tang, or Chai-Hu-Jia-Long-Gu-Mu-Li-Tang) was associated with lower overall mortality (aHR = 0.42, 95% CI: 0.21–0.85, *p* = 0.02) compared to patients who did not receive TCM (Table S1). Based on the high prevalence of depression and the increased risk of death in prostate cancer patients, our research can act as a bridge between clinical treatment and basic research, as well as serving as a reference for clinicians.

One of the characteristics of TCM theory is that TCM contains multiple formulae in a single prescription, which can effectively provide personalized medicine and reduce side effects. However, this prescription model also increases the difficulty of independently analyzing the clinical efficacy of a single formula. Previous studies have shown that different Chinese herbal compounds play different roles in the care of patients with prostate cancer. For example, Wen-Dan-Tang can effectively alleviate the anxiety associated with insomnia by regulating the performance of the ghrelin receptor in the hypothalamus and the concentration of leptin in the brain [19,20]. Chai-Hu-Jia-Long-Gu-Mu-Li-Tang can reduce the symptoms of hypogonadism caused by castration therapy, including sexual dysfunction, hot flashes, night sweats, and insomnia [21]. It also reduces the expression of cancer cell proteases, such as tumor-specific matrix metalloproteinases-2 and -9 and impedes the proliferation of prostate cancer [22]. The primary role of Jia-Wei-Xiao-Yao-San is also to alleviate the symptoms of decreased gonadal hormones, particularly mental symptoms, such as depression, sleep disorders, and irritability [23]. Jia-Wei-Xiao-Yao-San can also assist the body in combating chronic stress symptoms and regulates glucocorticoid receptors [24]. Gan-Mai-Da-Zao-Tang can alleviate the symptoms of depression and regulate monoamine neurotransmitters in the brain [17]. Suan-Zao-Ren-Tang is one of the prescriptions most commonly prescribed for insomnia and depression by TCM physicians in Taiwan, which can improve sleep quality, reduce nightmares, and improve quality of life [15,25]. 

In this study, we found that TCM formulae for depression could reduce the total mortality of patients with prostate cancer. We conclude that TCM compound might not directly kill cancer cells, but help patients have a better mood to receive modern medical treatment, including radiation treatment, chemotherapy, and castration therapy. In addition, TCM may reduce the side effects of castration therapy to improve patient’s acceptance. As part of a modern care system, TCM could alleviate the symptoms of patients and promote patients to receive more medical treatment [14,15].

In this study, we selected the top six drugs most commonly used by Chinese medicine practitioners to treat patients with depression and prostate cancer based on the high prescription of TCM physicians and the specificity of depression. However, we also found that TCM physicians did not use some antidepressant TCM formulae that have proven useful in clinical trials. For example, a systematic review and meta-analysis study found that Chai-Hu-Shu-Gan-San can effectively reduce the severity and frequency of depression [26]. However, Taiwanese TCM physicians rarely use it to treat patients with depressive prostate cancer. This phenomenon shows that TCM theory and practice have regional differences, and reveals that TCM is a progressive discipline. We encourage TCM physicians to have a wide acceptance of new medical knowledge. 

Past research has indicated that patients who receive TCM treatment may have greater health awareness and willingness to receive medical care [27]. Comparing only patients with and without TCM may lead to the inclusion of unknown confounding factors that influence the results. Therefore, we used two control groups in this study: patients who did not receive any TCM treatment and patients who received TCM other than spirit-quieting TCM. The baseline variable was compared between the spirit-quieting TCM group and the other two comparison groups in Table 1. No significant difference was found except for liver cirrhosis. We also used the Cox proportional-hazards model to calculate the risk of death and correct for demographic and medical characteristics to reduce the effect of confounders on the results. Unfortunately, we could not perform propensity score matching to reduce indication bias because the number of samples was insufficient. Studies with larger samples and longer follow-up periods are warranted in the future.

This study had some limitations. First, we used the diagnosis code for depression in the ICD-9-CM as a standard, but the database did not contain detailed medical records or interview records and, therefore, we could not measure the severity of depression. Furthermore, all patients enrolled in the study were treated with antidepressants, but we were unable to assess any psychological counseling and group therapy that was not covered by National Health Insurance. Second, the database did not contain information on the staging of prostate cancer, prostate-specific antigen levels, or Gleason scores. Therefore, we used the initial treatments for prostate cancer to estimate the cancer stage at diagnosis according to previous literature [11,28,29]. There was no information about cancer stage in LHID2000. The percentage of each stage at the diagnosis of prostate cancer came from Taiwanese Cancer Registry. The data in LHID2000 was de-identified, so we could not correlate it to Taiwanese Cancer Registry. Alternatively, we used the initial treatment strategies to estimate the stage at diagnosis. Most of the treatments for each stage of prostate cancer were reimbursed by Taiwanese National Health Insurance (NHI), and physicians should stick to the guidelines and indications of treatments announced by NHI to get the reimbursed medical fee. Patients who received the same treatment may differ in terms of symptoms. Third, this study was limited to TCM treatments covered by National Health Insurance, including Chinese herbal medicine and acupuncture; Tai Chi, meditation, and aromatherapy were not included in this study. There exists a possibility of patients receiving TCM outside the purview of National Health Insurance. Finally, some of the estimates are rather imprecise, seemingly on account of limited sample size.

## 5. Conclusions

In conclusion, in this broad national retrospective cohort study, we found that TCM for depression might be associated with a lower risk of overall mortality of prostate cancer with depression during a median follow-up period of 6.2 years. Additional randomized controlled trials are warranted to verify the clinical outcomes of TCM treatment for patients with prostate cancer.

## Figures and Tables

**Table 1:** Demographic and medical characteristics of prostate cancer patients according to the use of traditional Chinese medicine (TCM).

Variables	Traditional Chinese Medicine for Depression No. (%)	Other Traditional Chinese Medicine No. (%)	Did not Use Traditional Chinese Medicine No. (%)	Chi-Square Test *p*-Value
**Total**	81 (100.00%)	53 (100.00%)	114 (100.00%)	
**Age at diagnosis**				0.4797
<60	3 (3.7%)	4 (7.5%)	5 (4.4%)	
60–70	13 (16.0%)	9 (17.0%)	10 (8.8%)	
70–80	20 (24.7%)	14 (26.4%)	38 (33.3%)	
≥80	45 (55.6%)	26(49.1%)	61 (53.5%)	
**Urbanization**				0.0966
Very high	44 (54.3%)	31 (58.5%)	60 (52.6%)	
High	22 (27.2%)	13 (24.5%)	17 (14.9%)	
Moderate	12 (14.8%)	7 (13.2%)	24 (21.1%)	
Low	3 (3.7%)	2 (3.8%)	13 (11.4%)	
**Insured amount (NT$)**				0.4154
Dependent	17 (21.0%)	8 (15.1%)	31 (27.2%)	
1–19,999	34 (42.0%)	27 (50.9%)	49 (43.0%)	
20,000–39,999	18 (22.2%)	9 (17.0%)	24 (21.1%)	
≥40,000	12 (14.8%)	9 (17.0%)	10 (8.8%)	
**Comorbidity**				
Diabetes mellitus	44 (54.3%)	29 (54.7%)	47 (41.2%)	0.1146
Chronic kidney disease	20 (24.7%)	12 (22.6%)	21 (18.4%)	0.5563
Cerebrovascular accident	52 (64.2%)	29 (54.7%)	54 (47.4%)	0.0669
Coronary heart disease	52 (64.2%)	30 (56.6%)	63 (55.3%)	0.4376
Heart failure	27 (33.3%)	12 (22.6%)	39 (34.2%)	0.2947
Liver cirrhosis	42 (51.9%)	30 (56.6%)	42 (36.8%)	0.0252 *
Hypertension	71 (87.7%)	43 (81.1%)	95 (83.3%)	0.5573
**Prostate cancer type**				
Localized or Locally advanced	15 (18.5%)	8 (15.1%)	23 (20.2%)	0.0681
Metastatic	51 (63.0%)	36 (67.9%)	74 (64.9%)	0.4013
Castration-resistant	15 (18.5%)	9 (17.0%)	17 (14.9%)	0.0779

Note: NT$: New Taiwan dollars, of which 1 US $= 31.1 NT$. * *p* < 0.05.

**Table 2:** Adjusted hazard ratio with a 95% confidence interval of overall mortality in a national prostate cancer cohort with depression.

Different Treatment	Crude HR (95%CI)	Adjusted Model 1 aHR (95%CI)	Adjusted Model 2 aHR (95%CI)
Patients did not receive traditional Chinese medicine	[Reference]	[Reference]	[Reference]
Patients who received traditional Chinese medicine for depression (spirit-quieting TCM)	0.41 (0.24–0.73)	0.46 (0.23–0.88)	0.42 (0.21–0.85)
Patients who received other traditional Chinese medicine	0.83 (0.42–1.66)	1.02 (0.53–1.96)	1.02 (0.52–1.99)

Note: TCM: traditional Chinese medicine. HR: hazard ratio. aHR: adjusted hazard ratio. Adjusted model 1: adjusted model, including age at diagnosis, urbanization, and insured amount. Adjusted model 2: adjusted model, including age at diagnosis, urbanization, insured amount, comorbidity disease, and prostate cancer type.

## Supplementary Materials

The following are available online at https://www.mdpi.com/2077-0383/8/2/218/s1, Table S1: The herbal composition of six formulae for the treatment of depression.
