# Supplementary material for: Spirit-Quieting Traditional Chinese Medicine May Improve Survival in Prostate Cancer Patients with Depression
Source: J Clin Med. 2019 Feb 8;8(2):218. doi: 10.3390/jcm8020218 (PMC6406565; doi:10.3390/jcm8020218)
Supplement: Supplementary file 1 [file jcm-08-00218-s001.pdf]

Table S1 The herbal composition of six formulae for the treatment of depression in the manuscript

Chinese Medicine Formulae	Herbal Composition
Wen-Dan-Tang<br>溫膽湯	Pinellia ternata (Thunb.) Breit., Citrus reticulata Blanco, Poria cocos (Schw.) Wolf, Glycyrrhiza uralensis Fisch., Bambusa tuldoides Munro, Citrus aurantium L., Zingiber officinale Rosc., Ziziphus jujuba Mill.
Tian-Wang-Bu-Xin-Dan<br>天王補心丹	Asparagus cochinchinensis (Lour.) Mer r., Panax ginseng C. A. Mey., Poria cocos (Schw.) Wolf, Scrophularia ningpoensis Hemsl., Salvia miltiorrhiza Bge., Polygala tenuifolia willd., Platycodon grandiflorum (Jaoq.) A.DC., Angelica sinensis (Oliv.) Diels, Schisandra chinesis (Turcz.), Ophiopogon japonicus (L.f.) Ker-Gawl., Platyclatus orientalis (L.) Franco, Ziziphus jujuba Mill. var. spinosa (Bunge) Hu ex H. F. Chou., Rehmannia glutinosa Libosch
Suan-Zao-Ren-Tang<br>酸棗仁湯	Ziziphus jujuba Mill. var. spinosa (Bunge) Hu ex H. F. Chou., Glycyrrhiza uralensis Fisch., A nemarrhena asphodeloides Bge., Poria cocos (Schw.) Wolf, Ligusticum chuanxiong Hort.
Jia-Wei-Xiao-Yao-San<br>加味逍遙散	Angelica sinensis (Oliv.) Diels, Poria cocos (Schw.) Wolf, Gardenia jasminoides Ellis, Mentha haplocaly Brio., Paeonia lactiflora Pall., Bupleurum chinensis DC, Glycyrrhiza uralensis Fisch., Atractylodes macrocephala Koidz., Paeonia suffruticosa Andr., Zingiber officinale Rosc.
Gan-Mai-Da-Zao-Tang<br>甘麥大棗湯	Glycyrrhiza uralensis Fisch., Trticum aestivum L., Ziziphus jujuba Mill.
